# A mixed methods evaluation of a shared electronic health record between general practice and community pharmacy

**DOI:** 10.1007/s11096-025-01972-6

**Published:** 2025-08-07

**Authors:** J. Fynn, M. Lamptey, G. Coote, M. J. Twigg, J. Newman, S. Lingard, M. Cooper, H. Nazar

**Affiliations:** 1 Health Innovation East, Shelford Bottom, Cambridge, CB22 3AD UK; 2 School of Pharmacy, University of East Anglia, Norwich, NR47TJ UK; 3 NHS Norfolk and Waveney Integrated Care Board, Norwich, NR12DH UK; 4 NHS England, East of England Region, Victoria House, Camlife, Fulbourn, Cambridgeshire, CB21 5XB UK; 5 National Institute for Health and Care Research Newcastle Patient Safety Research Collaboration, Newcastle University, Newcastle Upon Tyne, UK; 6 School of Pharmacy, Newcastle University, Newcastle-Upon-Tyne, NE1 7RU UK

**Keywords:** Community pharmacy, Electronic health record, Primary care, Summary care record

## Abstract

**Introduction:**

Integrating community pharmacies into primary care via digital infrastructure is crucial to enhancing continuity, coordination, and safety of care. Historically, community pharmacies have not had full access to general practice electronic health records (EHRs), limiting their ability to provide informed interventions. The introduction of shared, interoperable EHRs has the potential to address this limitation and redefine the clinical role of community pharmacists.

**Aim:**

This study aimed to evaluate the feasibility, acceptability, and impact of granting community pharmacies read-and-write access to a shared EHR system (SystmOne) across selected sites in the East of England.

**Method:**

A 12-month mixed-methods pilot (Jan–Dec 2023) was conducted using an explanatory sequential and convergent approach. Data were collected from 35 community pharmacies and 31 general practices via activity logs, surveys, and semi-structured interviews. Descriptive statistics was used to analyse quantitative data and thematic coding used for analysing qualitative data. Data was then integrated to evaluate service delivery, communication, and user experience.

**Results:**

Thirteen community pharmacies actively used the EHR, documenting over 19,000 appointments and 16,000 clinical entries. Usage varied, with barriers including workload, technical complexity, and duplicated documentation requirements. However, users reported improvements in patient safety, interprofessional communication, and service efficiency. Appointment booking and task-sharing functions fostered collaborative working, while access to real-time clinical information supported clinical decision-making. Training support, trust between sectors, and policy alignment were identified as critical enablers for system uptake.

**Conclusion:**

Providing community pharmacies with read-and-write access to a shared EHR is feasible and contributes to safer, more integrated patient care. Improved communication, clinical documentation, and task delegation between pharmacists and general practice staff represent a major shift in digital collaboration. However, successful scale-up requires investment in interoperability, national IT infrastructure alignment, and streamlined reimbursement processes to prevent duplication of effort. These findings support the evolving clinical role of community pharmacists and suggest that integrated digital systems are essential to realising the full potential of community pharmacy in the modern NHS to improve patient care.

**Supplementary Information:**

The online version contains supplementary material available at 10.1007/s11096-025-01972-6.

## Impact statements

- Shared EHR access empowers community pharmacists to contribute more effectively to patient care by enabling real-time documentation, improved clinical decision-making, and perceived safer service delivery.
- Integrated digital systems between general practice and community pharmacy can enhance communication, reduce duplication, and streamline referrals—improving efficiency and reducing administrative burden for both sectors.
- Successful implementation of EHR in community pharmacy settings requires coordinated training, system interoperability, and alignment with national service reimbursement processes to minimise workload duplication and maximise engagement.

## Introduction

Collaborative and integrated working, especially between general practice (GP) and community pharmacy, was been recognised to reap many benefits, including; resource efficiency, service delivery continuity and improved quality and safety of patient care [1]. A recent realist review found that digital integration where general practitioners and community pharmacies shared information and electronic health records, there was an improved and common understanding of the patient issues and needs. This is reported to avoid duplication of effort, address gaps in care and enable shared decision-making [2].

The COVID-19 pandemic has demonstrated the importance of interoperable, connected digital systems across services [3]. Obtaining access to reliable information was important for tracking supplies and deliveries related to the COVID-19 vaccination programme. The pandemic raised awareness of IT functions for enhanced delivery of essential services (*e.g.,* medication planning, prescribing and dispensing between pharmacies and general practices) and advanced services, including vaccinations [3].

Patient Medication Records (PMR) are the standard digital platforms that are used within community pharmacies in England to record limited patient information (*e.g.,* demographics, contact details, allergies) history of medicine supply and delivery of medication and/or clinical interactions/interventions. However, these records do not form part of a patient’s primary care clinical record but are standalone and are not interconnected between individual pharmacies [4]. This means any given pharmacy will only have records of the medicines and/or interventions/services they have provided within their pharmacy or pharmacy chain. The PMR also does not include other information about medical conditions, new diagnoses, biochemical results, etc. unless the pharmacist collects this information from the patient and enters it themselves. This is because community pharmacy has not historically been considered part of primary care [5]. International terms for PMR include: pharmacy computer system, pharmacy information system and pharmacy information management system [5]. The functionalities of each system will inevitably vary depending on the intricacies of the healthcare contexts within which they are used.

Community pharmacies also used a myriad of other standalone IT platforms for the purpose of recording and claiming for services, *e.g.,* Pharmacy Services (Cegedim), PharmOutcomes (EMIS Pinnacle), HxConsult (Positive Solutions) and Sonar (Sonar Health). Recent national developments have seen a link to general practice systems by GP Connect (otherwise termed ‘middleware’) however, this currently relies on general practices agreeing to such connectivity [4].

In 2016, community pharmacies were given access to patients’ Summary Care Record (SCR) alongside other professions across the health and care system. Around 96% of patients in England have a SCR, which provides key clinical patient information (Box 1) [6] sourced from their general practice electronic patient record. A community pharmacist can use the information in the SCR (if a patient has consented) to check allergies to prevent prescribing errors, eligibility for services (*e.g.,* free flu jab), and current medications prescribed for emergency supply purposes [7]. The use of SCR in community pharmacies has had clear benefits for patients and staff as it has led to fewer referrals to other NHS care settings, a reduced need for phone calls to general practices, fewer prescribing errors, reduced patient waiting times and improved service for patients [8].

**Box 1:** Information recorded in the Summary Care Record [6]

The minimum data recorded includes: • Current medication • Allergies and details of any previous reactions to medicines • The name, address, date of birth and NHS number of the patient Additional data could include (unless patients have declined consent for this to be shared): • Details of long-term conditions • Significant medical history • Specific communications needs	

Despite the positive impact of access to the SCR, the need for community pharmacists to have full read-and-write access to patient’s healthcare records remains. In 2022, the Professional Record Standards Body (PRSB) published a report that stated due to the nature and complexity of pharmacists’ involvement in patient care and treatment, full access is fundamental and will result in an improvement to patient safety and care [9]. This report highlighted that community pharmacists are often the first point of contact for patients regarding medicine-related enquiries and play a vital role in supporting patients with long-term conditions. Additionally, the PRSB hoped that this level of access would result in improved communication between pharmacy teams and other healthcare professionals, including general practice, as well as reducing the chances of errors being made and providing an audit trail [9].

The policy context underpinning community pharmacy, the potential for improved communication and the impact on safety have led NHS England, East of England region to explore providing read-and-write access to the primary care clinical record held in general practice. This study aims to describe and evaluate community pharmacy using an integrated clinical electronic health records (EHR) system (SystmOne) in selected sites across the East of England as part of a pilot. The use of an integrated clinical system was intended to enable functionality currently unavailable to community pharmacies such as recording directly to the patient care record in real time (write access); read access to the primary care clinical record held in general practice, with patient consent; use of templates integrating decision support tools and information from the EHR and an integrated appointment booking system.

## Aim

This study aimed to evaluate the feasibility, acceptability, and impact of providing community pharmacies with read-and-write access to a shared EHR (SystmOne) used in general practice across selected sites in the East of England.

## Method

The 12-month pilot data collection period was from 1st January 2023 to 31st December 2023. Information about the context of the pilot site is provided in the supplementary file 1, this includes details about the intricacies of the healthcare system in England. A project team was convened which comprised of independent health service researchers [HN & MT], a team of evaluators [JF, ML, GC], implementation team [JN, SL] and clinical lead [JN]. The team were responsible for the design, conduct and oversight of the evaluation and production of dissemination materials. A logic model was developed in the design stage and used to guide the evaluation (Supplementary File 2).

A multi-stage mixed-methods evaluation [10] was conducted to investigate the objectives outlined in Table 1.

**Table 1:** Study objectives and associated data collection strategies

Objective	Data collection	Time point
To assess how and to what extent community pharmacies utilised the EHR system	Activity data extracted from SystmOne	Pilot end
To explore the perceived impacts and acceptability of this new way of working for community pharmacy and general practice	Surveys Staff interviews	Baseline and final months of pilot
To identify the issues, considerations and improvements for future deployments of EHR systems in community pharmacy		

A combination of explanatory sequential and convergent approaches was used [10]. The former involved surveys and service data informing the interviews at cross-sectional time points, *i.e.,* baseline and through the pilot. The convergent approach meant that the iterative stages of data collection and analysis occurring longitudinally through the pilot informed future data collection and analysis to produce the results. Findings have been integrated through a narrative where quantitative and qualitative findings have been weaved together to answer the research objectives [10].

Reporting of the study has followed the Revised Standards for Quality Improvement Reporting Excellence (SQUIRE 2.0) guidelines [11].

### Data collection

#### Activity indicators

Participating community pharmacies were asked to record clinical patient interactions using the bespoke record templates on SystmOne. Prior to the commencement of the pilot, a list of key activity indicators was agreed by the project team. A summary of the activity indicators is provided in supplementary file 3. The final data set was centrally extracted by the back-end management of SystmOne, exported into Microsoft Excel and sent to the research team on 18 January 2024.

#### Surveys

Three surveys were developed by the project team with questions aligning to the research objectives and key performance indicators. Surveys were not piloted. They were conducted at different time points during the pilot:

(i) Baseline survey to understand the communications, interactions and relationship between community pharmacies and associated general practices was sent to all community pharmacies opting to participate in the pilot (n = 35) and associated general practices (n = 31) (nature of association is described in the pilot context provided in supplementary file 1). The baseline survey was conducted between November 2022 and January 2023; responses were received between November 2022 and May 2023. This included 23 questions about pre-pilot communication and integrated working between community pharmacy and general practices, current practices relating to referrals, and expectations for the pilot.
(ii) *Follow-*up surveys (one for community pharmacy and one for GP practices) were sent at the end of November 2023 to the participating community pharmacies (n = 13) and the associated general practices (n = 19) that had been identified as having used their EHR units. Survey responses were received between November 2023 and January 2024. The survey contained 24 questions for community pharmacy and 21 questions for GP practices. The survey aimed to capture perceived impact and experience of the shared EHR relating to communication and relationships between general practice and community pharmacy; usability, engagement and impacts of sending and receiving referrals, the appointment rota and task function; and staff satisfaction with training and insights on patient experience.
(iii) Follow-up survey sent to non-participating pharmacies (n = 20) that had registered to take part in the pilot but did not download the allocated SystmOne unit or use the unit once downloaded. This survey was sent to pharmacies in November 2023, with the last response received in December 2023. The survey asked a single multiple-choice question with an optional open-text field to identify the reason(s) for the community pharmacy not actively using the EHR.

Individuals completing the surveys were asked to respond representing the views of their organisation (pharmacy or general practice) rather than their individual views. Surveys retained the identification of the organisations to enable us to identify people to invite for interview, and to facilitate follow-up questions to support analysis and enable comparison between baseline and follow-up and between associated pharmacies and general practices, where possible. This also allowed us to follow up with organisations to maximise survey response rates.

#### Interviews

Interviews were conducted with community pharmacy and general practice staff at two time points following the baseline and follow-up surveys. Purposive sampling was used to identify community pharmacies that had responded to the survey and based on their level of engagement with the pilot (high, medium, low), with the associated general practices being selected accordingly. General practice staff and community pharmacists within the selected sites were emailed to invite them to participate in an interview and were provided with a participant information sheet and consent form.

Initial interviews with community pharmacies (n = 7) and GP practices (n = 3) were conducted between July–September 2023. These aimed to understand factors influencing the early stages of the pilot. Follow-up interviews with community pharmacies (n = 5) and GP practices (n = 2) were conducted between January–February 2024, these were intended to explore how the pilot worked in practice, benefits and challenges, and suggestions for improvements that could help inform future scale-up. Semi-structured topic guides were developed for each round of interviews and were informed by the objectives of the evaluation, the key performance indicators and sought to gain more in-depth understanding of concepts explored within the surveys.

Interviews were conducted on Microsoft Teams or by phone and were recorded, transcribed verbatim and reviewed for accuracy prior to analysis. Microsoft Word documents of the transcripts were uploaded into Nvivo 14, a software for managing data for qualitative data analysis.

### Data analysis

Descriptive statistics were used to analyse and report the EHR activity indicators and the quantitative data from the closed survey questions.

Qualitative data from both the interviews and open questions in the surveys were analysed using thematic coding [12]. Initial codes were identified inductively through familiarisation with the data by GC and JF. A sample of transcripts were initially coded, comparing and agreeing any refinements and adding clear descriptors for codes to agree the coding framework that was then applied to all data sources. This was then shared and agreed through discussion with the wider evaluation team (MT, HN) and project team (SL, JN). GC and JF used notes function in NVivo and regular review meetings to cross-check coding and discuss any questions arising to ensure coding was documented, transparent and consistent. Codes were deductively mapped to the research objectives (namely objectives 2 and 3 which formed the overriding themes) towards developing subthemes. Qualitative data collection was ceased when data saturation was achieved across the codes generated (code saturation and stabilisation of the codebook) and generated subthemes from interviews and the open responses from surveys, with new interview data proving redundant. Supplementary file 4 includes the themes, subthemes and codes.

The findings have been woven together and presented as a narrative to address each objective. Objectives 2 and 3 have been presented as the descriptive themes with interpretive subthemes and illustrative quotes and/or survey responses.

### Ethics approval

Institutional ethical approval for the study was obtained from the Faculty of Medical Sciences ethical review committee (Ref: 32129/2023).

## Results

### Objective 1: How and to what extent community pharmacies utilised the electronic clinical record system

**Fig. 1:**
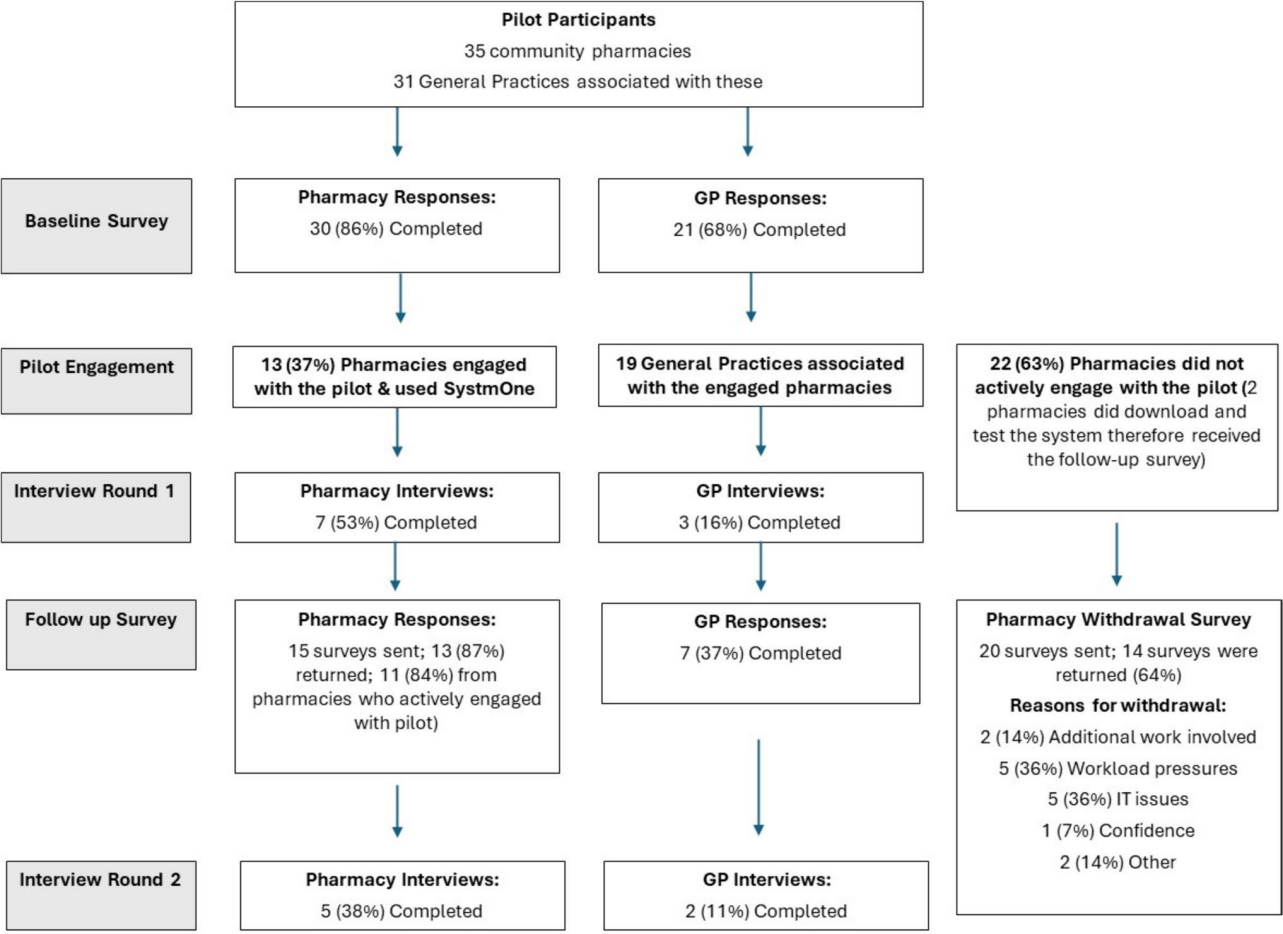
Overview of the data collection and sample included in the results

There were 35 community pharmacies which were associated with 31 GP practices that enrolled to participate in the pilot. However, only 13 (37%) of these pharmacies engaged with the SystmOne platform. These pharmacies were linked with 19 GP practices. The flow chart (Fig. 1) provides an overview of the data collection and sample included in the results.

Twenty-two community pharmacies did not actively use SystmOne, defined as those who registered but did not download the system (n = 20) or downloaded and tested but did not use it (n = 2). The 20 who did not download the system at all were sent a follow-up survey to ask their reasons for not engaging in the pilot. The primary reasons for dis-engagement (n = 14, 64% response rate) were related to workload pressures and potential increases in work that participation would involve, as well as IT issues (Fig. 1).

#### Pharmacy appointments booked and reported

All 13 pharmacies received direct appointment bookings from GP practices using SystmOne, with numbers ranging from 8 to 14,646 appointments booked, with a total of 19,314 appointments made across all pharmacies. Of those booked appointments, the total number reported as Did Not Attend (DNA) was 1968 (10%).

#### Clinical consultations and information recorded by pharmacies

Table 2 shows clinical consultations and information recorded by pharmacies across two categories: Essential and Advanced Services. Essential Services are defined as those which community pharmacies must provide under the community pharmacy regulation. Whereas Advanced Services are priced services that community pharmacies can choose to provide if they meet the requirements set out in the Secretary of State Directions [13].

**Table 2:** Consultations and clinical information recorded by pharmacies

	Pharmacies n(%)	Consultations and clinical information recorded		
		n	Mean per community pharmacy (± SD)	Range
Essential services	13 (100%)	1,643	126 (281)	1–1,023
Advanced services	13 (100%)	14,716	1,132 (674)	1–4,524

All pharmacies recorded consultations and clinical information pertaining to both essential services (*e.g.,* providing a discharge medication review service) and advanced services (*e.g.,* providing a consultation after receiving a referral from urgent and emergency care).

#### Task functions used

Nine (69%) pharmacies utilised the EHR task functions to request actions by GP practices. A total of 90 GP consultation follow-ups were requested, of which 88 (97%) were reported as actioned by GP practices. Three pharmacies sent tasks reported as ‘other’ to GP practices, of which 14 (82%) were reported as actioned. Four (31%) of the GP practices sent medication queries to pharmacies, of which half were recorded as actioned by the pharmacies. One GP practice sent 20 ‘other’ tasks to the pharmacy, 100% (n = 20) of which were reported as actioned.

### Survey responses relating to usage of SystmOne

Table 3 shows the number of pharmacies and GP practices who indicated they had used the EHR appointment task function across a range of services. Table 4 shows referrals for a range of services that were reported in the survey to have been sent to (by GP practice, n = 7) and received by pharmacies (n = 13) using the task functionality in SystmOne.

**Table 3:** Number of pharmacies and general practices reporting use of the SystmOne appointment rota for referral to pharmacy services

	Blood Pressure Service Check	Contraceptive Services	NMS*	Other
	N (%)			
Pharmacies receiving referrals from general practices	7 (53.8)	8 (61.5)	3 (23.1)	2 (15.4)
General practices sending referrals to pharmacies	6 (85.7)	5 (71.4)	6 (85.7)	4 (57.1)

^*^NMS: New Medicines Service

**Table 4:** Survey responses from pharmacies and general practices showing the use of SystmOne to support the delivery of Essential and Advanced Services

	Pharmacy(n,%)	General practice (n,%)
Hypertension case finding	9 (69.2)	5 (71.4)
Contraceptive services	8 (61.5)	3 (42.8)
New medicines service	4 (30.7)	4 (57.1)
Discharge medicines (DMS)	4 (30.7)	NA
Prescription Queries	6 (46.2)	5 (71.4)
Other	0	1 (14.2)

### Objective 2: The perceived impacts and acceptability of this new way of working for community pharmacy and general practice

#### Impact on the quality and safety of patient care

When asked if SystmOne had improved the delivery of patient services and care, nine (69%) community pharmacy respondents strongly agreed or agreed; two (15%) respondents disagreed; and another two (15%) neither agreed nor disagreed. Similarly, when asked if the clinical system had improved clinical decision making, seven (54%) respondents strongly agreed or agreed; with just one (8%) respondent disagreeing; and the remaining five (38%) neither agreeing nor disagreeing. These improvements were attributed to increased access to patient information, allowing pharmacies to have a more holistic view of the patient, resulting in safer practice (from survey open-text fields).

Pharmacy respondents highlighted in the interviews:

*“… being able to see previous consultation notes from other healthcare professionals which help guide our consultation […]. We are also able to get a holistic view of the patient e.g. details of recent hospital admissions and investigations [...] all these factors are the building blocks of a patient consultation. ” (P08-interview)*

One GP interviewee commented on improved patient safety:

*“Ability to share information about medication errors, adverse drug reactions, and other safety concerns between practices and community pharmacies can help improve patient safety. This can lead to better monitoring of high-risk medications, improved communication between providers, and better management of patient medications.” (GP19-interview)*

## Impact on efficiencies and staff capacity

When asked if having access to the appointment rota was beneficial to their GP practice or pharmacy, five (71%) GP respondents and five pharmacy respondents (38%) agreed or strongly agreed. While only three (23%) pharmacy respondents strongly disagreed, five (38%) indicated they neither agreed nor disagreed. Respondents who agreed or strongly agreed stated that the introduction of an appointment system had improved capacity for practices and helped pharmacies manage their workload (open-text fields).

This was corroborated with GP respondents commenting:

*“SystmOne allows minor ailments appointments to be booked directly into local pharmacist clinic which saves so much time for the surgery and allows the surgery to focus on urgent and complex patients.” (GP02-interview)*

*“Reduces time spent completing admin for our own staff. Clinicians can see the Pharmacy consultation immediately after it has taken place.” (GP11-survey)*

Pharmacy interviewees relayed how the shared appointment rota allowed work to be more efficient and improved organisation and management.

*“It significantly reduced our time contacting GPs. I’m not looking for email addresses, I’m not sending it to an old practice manager. I get it tasked to the right team and then it gets actioned.” (P04-interview)*

### Impact on patients and patient access

Although feedback was not collected directly from patients, staff commented on the positive feedback they had received from patients. This focused on the improved access to care. Both GP practice and pharmacy staff mentioned patients commenting on being able to get an appointment more quickly. With one practice commenting:

*“Patients have been delighted with the service offered by the Pharmacy and particularly like that we can book them an appointment directly.” (GP11-survey)*

While pharmacies reported:

*“And those patients were really grateful that they could just walk into the pharmacy and have this consultation, and they were booked in at a time and they were getting seen.” (P35-interview)*

A number of pharmacy and GP respondents also stated that pharmacies having access to SystmOne had led to improvements in the service users’ experience:

*“Patients have also had satisfaction that our treatment decisions have been documented in their GP notes, especially in instances where we have asked them to monitor symptoms and return if needed, they felt they would not need to start all over again with their consultations.” (P08-interview)*

*“Clearly demonstrated significant improvements in patient access, ease of interface and fostering excellent working relationships, it should be extended to other pharmacies to level the playing field and bring those benefits to more practices, pharmacies and patients.” (GP28-interview)*

However, some respondents shared reflections that described less positive patient experiences. These tended to relate to their overall experience and expectations of using the community pharmacy rather than specifically the patient experiences of this pilot.

### Impact on integration, relationships, and communication

Eight (62%) community pharmacists and six (86%) GPs either agreed or strongly agreed that the introduction of SystmOne had improved relationships between their organisations. Only two (15%) pharmacy respondents and one (14%) GP strongly disagreed or disagreed that relationships had been improved. Similarly, seven (54%) community pharmacies and six (86%) GPs either agreed or strongly agreed that the introduction of SystmOne had improved communications between their teams.

When asked what the pharmacies and GP practices had gained from pharmacies having direct access to the EHR, responses often included reflections on better understanding, continuity of care, improved communication, collaboration and relationships across teams (open-text fields).

For example, several pharmacy representatives reflected in their interviews on improved collaboration:

*“Our relationships with our GP surgeries are stronger and we are working together better than ever before, we have formed new relationships with surgeries who previously were reluctant to engage as they felt using community pharmacy services increased their admin burden.” (P08-interview)*

While GP practice reported:

*“It’s not just the IT platform, it’s because there is an IT platform, it supports communication across organisations and it supports the development of that two-way communication between different organisations and building of trust, and I think that’s the key benefit that we’ve seen.” (GP25-interview)*

However, one pharmacy interviewee highlighted that engagement from both sides was required to ensure access to the EHR was successful:

*“There is massive potential for this if all parties are involved and engaged i.e. the pharmacy and the surgery. However, in our case we have not had any referrals or messages sent through the pilot SystmOne for about 4 months now.” (P30-interview)*

### Confidence in pharmacies using the clinical system

Although some participants felt confident using the system soon after its introduction; others suggested that they needed time to become confident, commenting that initially the system seemed complicated and different from what they were used to:

*“It’s not difficult to learn. It may seem a bit complicated to start with […], I didn’t think I’d find it difficult to work so it is pretty straightforward after about four or five examples.” (P28-interview)*

### Perceived benefits

Findings from the interviews also highlighted various themes relating to staff perceptions of the acceptability of this new way of working. These closely aligned with beneficial impacts previously described, for example improved communication, strengthening of relationships, collaborative working and improved patient care.

*“Obviously it’s the collaboration that I really, really think is really great about this pilot is that it will really kind of embody pharmacy within GP practices and working collaboratively with them.” (P35-interview)*

The pilot itself was also described as an opportunity to showcase how the current ways of working and patient care could be improved. Some pharmacy and practices indicated their acceptance of this new way of working by highlighting either the need for continued use or the adoption by others in the future.

*“This should be standard of care. Any alternative outcome is to knowingly deny patients the opportunity for the safest, most effective treatment available to them. Discontinuation of the service, or the refusal of a health system to adopt shared EHR access including community pharmacies, would be a disservice and a risk of harm to patients.”* (GP28-interview)

*“I think unreservedly, we wouldn’t go back. We are very keen to keep SystmOne as the combined platform going forward, and we would really encourage community pharmacies to explore this because I think it enables the policy agenda of working together to support patients. And so we would highly recommend engagement with this.” (GP25-interview)*

### Objective 3: The issues, considerations and improvements for future deployments of EHR systems in community pharmacy

Some barriers were identified relating to feasibility of implementing and using the EHR. This includes the barriers identified by the 14 community pharmacies who didn’t implement or utilise SystmOne but responded to the follow-up survey.

#### Barriers to SystmOne implementation

Of the 22 pharmacies that had not downloaded or utilised their allocated SystmOne unit, 14 responded by survey sharing the reasons for lack of engagement. As previously noted IT/Technical issues and staff capacity were highlighted. Pharmacies provided the following reasons for not mobilising SystmOne:

*“Staff issues, as locums are just at the pharmacy for the day and will not use the system. Short staffed so the team just did not have time to assist. We cannot find a regular Pharmacist that will be able to provide the attention needed to benefit from the system. Also concerned at downloading external software to our computer.” (P23-interview)*

*“The system itself is something we are not used to. With out the option of repetitive practice it was difficult to get to grips with. Also when we did get it up and running the Surgery were unable to use it as they only had one member of staff able to configure the appointments their end.” (P07-interview)*

#### Barriers to SystmOne utilisation

Some pharmacy respondents could see the benefits of using SystmOne but had not been able to comprehensively engage for a number of reasons, including minimal/lack of GP practice engagement, capacity within the pharmacy, conflict with existing required community pharmacy IT systems and patient consent issues.

*“I attended a meeting with the GPs, with their monthly meeting explaining to them how this would benefit them in terms of their minor ailments that we can do the triaging system or just doing the minor consultation. But I don’t think they booked more than 10 appointments since we started.” (P28-interview)*

*“Great system to have but would like to have seen better support to help drive the pilot in terms of getting GPs on board.” (P35-survey)*

In line with national requirements, the engaged pharmacies still had to use nationally authorised IT platforms to claim service payments which meant a duplication of recording on different platforms:

*“When we were doing the hypertension service […] we have to make four entries. So we have the entries on our PMR system, we had to make the same entry on SystmOne, then we have to make the same entry on PharmOutcomes. And then the same entry on Emis. So for one piece of work, we were having to do four different entries, which makes absolutely no sense. I think, I think that will be a big barrier going forward.” (P29-interview)*

Records access and consent was also identified as a barrier by a few pharmacies:

*“Where the patient has consented, it is easier to check their history. However, many patients have chosen to opt out and as pharmacists, we cannot see their history. In almost all cases, patients are not aware as to why they had opted out.” (P29-interview)*

#### Enablers to SystmOne utilisation

Despite the potential barriers highlighted, key themes emerged from surveys and interviews relating to factors that enabled the utilisation of SystmOne in a community pharmacy setting, such as training and on-going support.

#### Training

Whilst some users found the system complex to learn, many felt supported throughout the learning process with just two (15%) pharmacies reporting that they were not satisfied with the training offered, seven (53%) respondents reporting that they were very satisfied or satisfied, and the remaining four (30%) providing a neutral response.

*“Training was offered at a time outside working hours not to impact the daily running of the pharmacy. Trainer was very helpful in taking time to make sure I was fully comfortable with using SystmOne.” (P24-survey)*

Others commented on the usefulness of bite-sized training videos that were issued following the initial training, reporting that they were a useful resource to refer to or use when setting-up certain functions.

*“What did help was additional videos and the clips I got sent through later on. And so we had a reference sort of point. So, if you got stuck anywhere, we could go back onto the videos and they’re really self-explanatory and we didn’t have a problem with that at all.” (P30-interview)*

Although training was identified as an enabler with pharmacies generally satisfied with both online and offline training videos, improvements in both the timing and delivery mode of training was highlighted.

*“Quite a hard system to navigate, felt with further training it was easy to use once you knew how but as there was a gap between training and initiation of pilot, we did struggle.”* (P35-interview)

The delivery of training was also identified as an area that could have been improved with a number of pharmacies suggesting that face-to-face training would have been better:

*“The clips were informative, but would be better if we could have used the training straight away and not months later […] I still think that it’s very different being shown something virtually, when it’s a brand new system, and someone actually physically in the pharmacy spending half an hour, an hour with you. I think that’s the key difference.” (P29-interview)*

#### Systems interoperability and integration

Several pharmacy staff highlighted the challenges of needing to duplicate work due to requirements to make claims on the nationally approved and mandated IT platforms. The potential for better interoperability of systems was mentioned by several staff:

*“The only way this is realistically going to happen is if you’re going to reach a situation where you’re going to be able to integrate IT systems within GP surgeries and pharmacy systems.” (P14-survey)*

#### Improvements in functions, templates and options

Practice and pharmacy participants mentioned the potential value in increasing the available tasks and functions. Some suggested that even with the current number of options, a staged approach to implementation would be beneficial to allow pharmacists time to increase their familiarity and confidence in using the system. One practice also discussed the potential for enhanced population health management functions that would be possible if patients could be registered longer term with community pharmacies, in a similar way to patient records in GP practices.

*“But what could be better in the future is that everyone that uses that pharmacy, if they could be just like everyone who uses my GP surgery, if they could be active people on the pharmacy SystmOne unit, that would allow a bit of population health management.” (GP25-interview)*

#### Exit plan from pilot

Several participants described their frustration over a lack of a clear exit plan for those pharmacies and GP practices that had engaged and invested time and resource in the pilot at the end of the twelve months:

*“Just that the exit plan is such a big disappointment. And I’m having to do a lot of work behind the scenes to find other ways to keep this going because we’ve built such a good thing with the PCN and it doesn’t actually just need to stop with my organization […] I will help others get it if I can.” (P04-interview)*

## Discussion

This pilot reports on a novel initiative where community pharmacies had read-and-write access to the EHR, making the pharmacies a data controller for their own data entries and demonstrating the potential collaborative approach to patient care across settings. Both community pharmacy and GP practice, made use of the system to assign tasks and make appointments in each other’s settings. Community pharmacy also made a considerable number of entries related to services and activities delivered in the pharmacy.

Surveys and interviews evidenced a high level of acceptability and confidence in using the integrated system. The findings highlighted benefits and impacts of this new way of working which support integration, such as enhanced communication, collaboration, understanding and trust between GP practice and community pharmacy. The improved access to information, the ability to book appointments, make referrals, and better relationships were all highlighted by both community pharmacists and GPs as having positive impacts for staff and patients.

Improved quality and safety of patient care were also recognised, attributed to better access to patient records and information. Writing directly onto the patient record meant that information pertaining to consultations, treatment or advice could be viewed in real-time by the patient’s GP without the delays inherent with sending consultation information via middleware or NHS mail which needs general practice review and acceptance.

Whilst the pilot has provided evidence of overall benefits, participants identified several enablers and barriers that had influenced adoption and engagement that warrant consideration for wider implementation and sustainability. A key concern was the duplication of data entry due to the multiple systems in use in community pharmacies to support various activities. It was also clear that some functionalities were better used by some pharmacies than others which warrants further attention. Training and awareness across both community pharmacy and GP practice could also have improved general engagement and use of the EHR since the potential of the system was not fully realised.

### Implications for policy and practice

Community pharmacy has long been considered an underutilised resource with highly trained personnel who are not using their clinical skills to maximum advantage for patients [14]. This is a timely concern in the United Kingdom as increasing numbers of existing pharmacists are becoming independent prescribers and from 2026, all newly qualified pharmacists will be able to prescribe [15]. In community pharmacy, there will be a need to appropriately record any prescribing activity within a patient record, so EHR access will be a necessity. This is of international import as the drive for prescribing pharmacists becomes of global interest.

There is also an increasing move to commission more advanced services from pharmacies in the UK [16], primarily to move workload from general practice and urgent care services to community pharmacy and to shift public perception to considering their pharmacy as the first point of contact for minor conditions. The UK government has outlined their intention to provide more patient care in primary care and community settings as part of the plan to reform the NHS [13]. The Darzi review of the NHS in England [17] concurs with this strategy, highlighting the further potential to expand the clinical role of community pharmacists and highlighting the accessibility for patients, particularly in areas of high deprivation.

Integrated, real-time digital communication between care settings and healthcare professionals will be crucial to ensure community-based care is coordinated, safe and effective. The capacity to record and share clinically significant information will also be crucial for effective service delivery and ensuring patient safety. Our study findings demonstrate that the shared access and functionality of an EHR can improve integrated working and service delivery across care settings. However, to introduce and ensure adoption of a new system within a clinical setting, like community pharmacy, requires a full understanding of how existing platforms, systems and ways of working will impact engagement and use. Functions and functionality need to be considered carefully to prioritise how these meet user needs and optimise engagement and potential efficiencies. A recent realist review also recommended that technology should support professional interaction and communication, allowing flexibility in what and how records are made within the system to promote patient safety and not undermine existing professional practice [2]. Existing implementation theories and frameworks can also be helpful to pre-empt, plan and mitigate challenges experienced during technology-supported change programmes in health and social care. The evidence-based NASS framework (**n**on-adoption and **a**bandonment of technologies by individuals and the challenges to **s**cale-up, and **s**ustainability) offers a map of possible domains of complexity to consider when planning, evaluating or reporting on a project or initiative involving a technology [18]. Designers, commissioners and implementers of wider EHR access across health (and social care) settings in future work, would benefit from structuring the approach using the NASS framework and/or adopting an implementation/behavioural science framework or theory to support programme implementation and evaluation.

### Strengths and limitations

The study captured data from multiple sources and perspectives allowing an in-depth evaluation of the feasibility, impacts and staff perceptions of community pharmacy having access and control of an EHR. The project team met iteratively throughout the pilot period to formatively review activity data, and practitioner (community pharmacy and GP) engagement. This allowed the evaluation to remain dynamic and responsive.

The sample size for the evaluation was limited by the number of community pharmacies and their associated general practices that actively engaged in the pilot. This was influenced by several factors including delays between training and receiving SystmOne units, and the lack of formalised agreements or funding for general practice involvement.

This study focused on just one EHR system, SystmOne, so it was not possible to explore the intricacies of interoperability and compare functionality. This may warrant further attention.

## Conclusion

This evaluation demonstrates it is feasible and acceptable to use an EHR clinical system in community pharmacy. Its adoption can realise benefits across integrated care delivery and potentially safer patient care.

Further work in this field should be carried out to trial the use of other EHR systems in community pharmacy and test the systems on a larger scale, exploring, the functional interoperability between general practices using one system and community pharmacies using another. The use of EHR systems for NHS prescribing in community pharmacy should also be investigated given the progress of pharmacy independent prescribing within the UK. This work should be underpinned with appropriate implementation theory and frameworks to ensure it is robust and systematic.

## Supplementary Information

Below is the link to the electronic supplementary material.

Supplementary file1 (DOCX 14 KB)

Supplementary file2 (DOCX 379 KB)

Supplementary file3 (DOCX 31 KB)

Supplementary file4 (DOCX 16 KB)

## Data Availability

Data have not been shared onto a repository but is available upon reasonable request.
