# Supplementary material for: A mixed methods evaluation of a shared electronic health record between general practice and community pharmacy
Source: Int J Clin Pharm. 2025 Aug 7;48(1):148–59. doi: 10.1007/s11096-025-01972-6 (PMC12823636; doi:10.1007/s11096-025-01972-6)
Supplement: Supplementary file 1 — Supplementary file1 (DOCX 14 KB) [file 11096_2025_1972_MOESM1_ESM.docx]

Context of the pilot site

Prior to the start of the pilot (November 2022) Primary Care Networks (PCNs) and individual general practices were invited to submit an expression of interest to the project team via the six regional Integrated Care Boards (ICBs). A total of 31 general practices (7 PCNs representing 20 general practices, and 11 individual general practices) who were using SystmOne were invited to participate.

As part of the pharmacy selection process, the project team identified community pharmacies who dispensed high volumes of prescriptions issued by the 31 invited general practices. This resulted in a shortlist of 67 community pharmacies who were invited to register for the pilot. A total of 43 pharmacies submitted an expression of interest, of which 40 went on to sign the Service Level Agreement (SLA). On receipt of the SLA, individual SystmOne Units were purchased on behalf of the 40 pharmacies. Six pharmacies withdrew prior to unit mobilisation, due to technical reasons. Only three of these six units could be repurposed for other interested pharmacies, leaving 37 pharmacies moving into the mobilisation stage. A further two pharmacies withdrew, resulting in a total of 35 pharmacies included in the pilot.
