# Supplementary figures and images for: A mixed methods evaluation of a shared electronic health record between general practice and community pharmacy
Source: Int J Clin Pharm. 2025 Aug 7;48(1):148–59. doi: 10.1007/s11096-025-01972-6 (PMC12823636; doi:10.1007/s11096-025-01972-6)

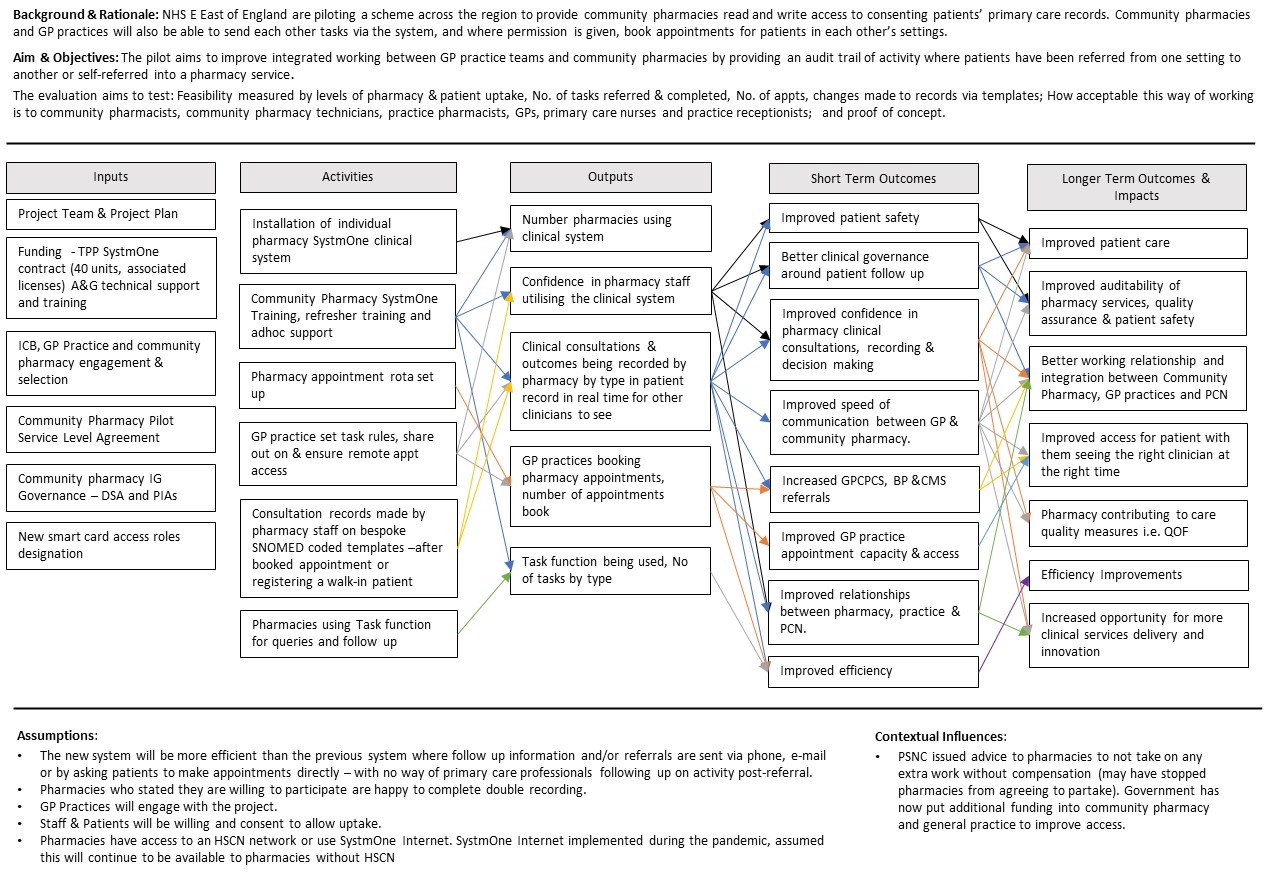

Supplement: Supplementary file 2 — Supplementary file2 (DOCX 379 KB) [file 11096_2025_1972_MOESM2_ESM.docx]
