# Supplementary material for: A mixed methods evaluation of a shared electronic health record between general practice and community pharmacy
Source: Int J Clin Pharm. 2025 Aug 7;48(1):148–59. doi: 10.1007/s11096-025-01972-6 (PMC12823636; doi:10.1007/s11096-025-01972-6)
Supplement: Supplementary file 3 — Supplementary file3 (DOCX 31 KB) [file 11096_2025_1972_MOESM3_ESM.docx]

**Supplementary File 3 Data relating to activities undertaken by pharmacies and general practices as part of the pilot**

| **KPI** | **Comments** |
| --- | --- |
| **Task Function** | |
| Number of Pharmacy to GP Tasks - Prescription Query | Pharmacies only have access to patient records with patient consent, therefore limiting the use of the task function for prescription queries. |
| Number of Pharmacy to GP Tasks - Prescription Query Actioned | Pharmacies only have access to patient records with patient consent, therefore limiting the use of the task function for prescription queries. |
| Number of Pharmacy to GP Tasks - Consultation Follow Up |  |
| Number of Pharmacy to GP Tasks - Consultation Follow Up Actioned |  |
| Number of Pharmacy to GP Tasks - Other |  |
| Number of Pharmacy to GP Tasks - Other Actioned |  |
| Number of GP to Pharmacy Task - Prescribing/Medication Query | Pharmacies only have access to patient records with patient consent, therefore limiting the use of the task function for prescription queries. |
| Number of GP to Pharmacy Task - Prescribing/Medication Query Actioned | Instances where pharmacies had actioned tasks but failed to mark as complete was found upon review, potentially impacting reporting of this metric. |
| Number of GP to Pharmacy Task - Other |  |
| Number of GP to Pharmacy Task - Other Actioned |  |
| Number of Pharmacy to GP tasks relating to NMS recorded | Only reported by one pharmacy. |
| Number of Pharmacy to GP tasks relating to NMS - Actioned | Only reported by one pharmacy. |
| **Commissioned Core Services** | |
| **Self Care/Sign Posting/Promoting Health Lifestyle** | |
| Number of clinically significant pharmacist Consultations Recorded (non CPCS) -Self Care Advice/ Sign Posting/Promoting Health Lifestyle | Recorded information from consultations deemed clinically significant but unlikely to make into the clinical patient record under normal circumstances due to no current mechanism or requirement to share this information |
| **DMS** | |
| Number of DMS consultations recorded on patient records | There was no specific DMS template developed as part of the pilot. |
| **Commissioned Advanced Services** | |
| Booked Appointments at GP Practice (Remote Booking) | Appointments were note coded by type, therefore these cannot be broken down by service. |
| Number of GP Booked Appointments that ended in a DNA | Appointments were not coded by type, therefore DNAs could not be distinguished. |
| **GP CPCS** | |
| Number of GP CPCS Consultations Recorded | We will look at pre pilot rates and provide these also alongside national activity. Without the system these consultation notes would have been sent by email and it would be dependent on the GP practice to put into the patient record. |
| Number of referrals to GP following GP CPCS consultation | High level of referral back to GP from Masters this has not been explored as practice could have been sending wrong patient type. |
| **CPCS (referrals from NHS 111)** | |
| Number of CPCS referrals recorded |  |
| Number of CPCS Minor Illness Consultations Recorded |  |
| Number of CPCS Urgent Supply Services Recorded | Good volumes of this service with the record going directly into the patient records rather than relying on NHS mail. |
| **Hypertension Case Finding** |  |
| Number of BP referrals from GP *(this would not necessarily by a booked appointment as different local referral routes)* | Measured by referral source report which relies on the pharmacy to tick referral or self-referral. |
| Number of BP services that were self-referral or pharmacy identified | Measured by referral source report which relies on the pharmacy to tick referral or self-referral. |
| Hypertension cases either GP referred or self-referred where a BP reading was recorded (does not include those BPs taken outside the BP Check Advanced Service ) |  |
| **NMS** | |
| Number of NMS referred by GP |  |
| Number of the NMS checks recorded on patient records |  |
| Number of NMS with intervention recorded | Under current processes, this data is shared via email only which is then uploaded manually by the GP practice. |
| **Contraceptive Medicines Service** |  |
| Number of Oral Contraceptive Repeat Consultations |  |
| **QOF Measures** | |
| Number of patient BP measures recorded (outside BP check service) - DM019 BP002 | These BPs will have been taken within consultations outside of the BP/Hypertension Service. |
| Number of patient BMIs recorded - OB002 |  |
| Number of patient smoking status recorded - SMOK002/5 |  |
| Number of smoking cessation or brief intervention services recorded - SMOK004 |  |
| **NOT REPORTED** | |
| **KPI** | **Comments** |
| Number of patient dissent to record access | This data was not extractable |
| Reason for refusal recorded at the time of refusal | This data was not extractable |
| Number of Pharmacy to GP tasks relating to GP CPCS recorded | A task category called GP CPCS was created but not used by pharmacists, and is reported within the total number of pharmacy to GP tasks and not reported separately. |
| Number of Pharmacy to GP Tasks relating to GP CPCS - Actioned | A task category called GP CPCS was created but not used by pharmacists, and is reported within the total number of pharmacy to GP tasks and not reported separately. |
| Number of Pharmacy to GP tasks relating to CPCS recorded | Any CPCS (NHS111) will be included in consultation tasks. |
| Number of Pharmacy to GP Tasks relating to CPCS - Actioned | Any CPCS (NHS111) will be included in consultation tasks. |
| Number of BP referrals directly book via remote booking | Appointments available to GP Practices were note coded by type |
| Number of NMS directly book via remote booking | Appointments available to GP Practices were note coded by type |
| Number of CMS directly book via remote booking | Appointments available to GP Practices were note coded by type |
| Number of the long term CMS services (Advanced and Tier 2 pilot) recorded on patient records | Not applicable. This service was a national pilot during the term of the IT pilot, therefore is not yet an established service. |
| Number of referrals to GP following CMS consultation | Not applicable |
| Number of Emergency Hormonal Contraception (EHC) recorded | Not applicable |
| Hypertension Case Finding - Number of urgent referrals (24 hour) to GP or other service (UEC or OOHs) recorded |  |
| Hypertension Case Finding - Number of non-urgent referrals ( 3 weeks) to GP recorded |  |
