# Supplementary material for: A mixed methods evaluation of a shared electronic health record between general practice and community pharmacy
Source: Int J Clin Pharm. 2025 Aug 7;48(1):148–59. doi: 10.1007/s11096-025-01972-6 (PMC12823636; doi:10.1007/s11096-025-01972-6)
Supplement: Supplementary file 4 — Supplementary file4 (DOCX 16 KB) [file 11096_2025_1972_MOESM4_ESM.docx]

| **Themes (Objectives)** | **Sub-Themes** | **Codes** |
| --- | --- | --- |
| 2. The perceived impacts and acceptability of this new way of working for community pharmacy and general practice. | Perceived Impacts of pilot | Patient Impacts |
|  |  | Staff Impacts |
|  |  | Efficiencies |
|  |  | Relationships & communications |
|  |  | Integration |
|  | Acceptability & appropriateness | Utilisation - Use of SystmOne Functionalities (appointments, clinical record, referrals, tasks, templates), use of services (GPCPCS, Hypertension, NMS, Oral contraception) |
|  |  | Acceptability |
|  |  | Functionality & usability - complexity, ease of use and settings |
| 3. The issues, considerations and improvements for future deployments of EHR systems in community pharmacy. | Barriers to implementation | Capacity & Workload |
|  |  | Funding & funding mechanisms |
|  |  | Lack of engagement - from different partners (i.e. GPs, Pharmacies) - relating to relationship, activities, integration & use |
|  |  | Lack of integration to existing systems |
|  |  | Limitation in training and support (availability, timing, accessibility) |
|  |  | Patient Consent |
|  |  | Prescription restrictions |
|  |  | Resistance to change |
|  | Enablers to implementation | Accessibility of SystmOne |
|  |  | Relationships & communication between practice and pharmacy |
|  |  | Training, support and instructions |
|  | Considerations for future |  |
